# Telehealth service use in people with cancer compared to people without cancer: analysis of Patient Experience Surveys in Australia

**DOI:** 10.1007/s00520-026-10851-3

**Published:** 2026-06-05

**Authors:** Huah Shin Ng, Ramya Walsan, Reema Harrison, Bogda Koczwara

**Affiliations:** 1 College of Medicine and Public Health, Flinders Health and Medical Research Institute, Sturt Road, Adelaide, SA 5042 Australia; 2 SA Pharmacy, Northern Adelaide Local Health Network, Adelaide, SA Australia; 3 Centre for Health Systems and Safety Research, Australian Institute of Health Innovation, Macquarie University, Sydney, NSW Australia; 4 Australian Research Centre for Cancer Survivorship, University of New South Wales, Sydney, NSW Australia

**Keywords:** Cancer, Perceptions, Telehealth, Health service, Australia

## Abstract

**Purpose:**

To compare the prevalence and characteristics associated with telehealth use in people with and without cancer.

**Methods:**

Cross-sectional study using data from the Australia’s Patient Experience Surveys (2022–2023 and 2023–2024) of all participants aged ≥ 25 years. Multivariable logistic regression analyses were performed to examine characteristics associated with telehealth use between cancer and non-cancer groups and to compare self-assessed health status, emergency department (ED) presentation and hospitalisations by telehealth use and cancer status.

**Results:**

Telehealth was used by over one-third of respondents with cancer (*n* = 797/2109, 38%) and one-quarter of respondents without cancer (*n* = 11,984/45,438; 26%). In both cancer and non-cancer groups, mental health conditions and other long-term health conditions were associated with higher odds of telehealth use, whereas being unmarried, uninsured, having a lower education level and a more recent survey were associated with lower odds of telehealth use. The odds of poor health status (adjusted odds ratio (aOR) = 4.29; 95% CI = 3.59–5.12), ED presentation (aOR = 2.69; 95% CI = 2.30–3.14) and hospital admission (aOR = 3.97; 95% CI = 3.41–4.61) were highest in people with cancer and telehealth use, followed by people with cancer who did not use telehealth and people without cancer who use telehealth, when compared to people without cancer and telehealth usage. Over 90% of people who used telehealth showed positive perceptions of the service regardless of cancer status.

**Conclusions:**

While over one-third of respondents with cancer utilised a telehealth service, the majority did not. Further research should investigate the barriers to accessing telehealth services to inform the development of health policy and strategies targeting people with greatest needs.

## Introduction

Telehealth or telemedicine involves a consultation with a health professional over the phone, by video conferencing or through other telecommunication technologies to deliver various aspects of healthcare such as diagnosis, treatment, prevention and monitoring [1, 2]. The provision of telehealth services has the potential to improve healthcare access due to geographical barriers, reducing the need for long distance travel and associated time and travel costs for patients living in rural and remote areas [3]. Telehealth service also offers an alternative way of delivering care to patients with chronic diseases that require regular monitoring and follow-up, providing some flexibility with appointment scheduling that fit patients’ preferences [3].

In Australia, patient access to telehealth services is supported by the ongoing Australian Government’s Medicare Benefits Schedule arrangements as part of the Government’s response to the COVID-19 pandemic [4]. There are specific geographical requirements in place such as the provision of service to a patient who is at least 15 km away by road from the healthcare provider at the appointment time or to a patient who is in a telehealth eligible area, although there are exceptions to this rule [5]. While telehealth has existed for a long time in Australia, its extent of use has been constrained primarily to rural and remote settings [6, 7]. Telehealth was also understudied until the COVID-19 pandemic, during which the restrictions on isolation and physical distancing rules have led to a fast evolution in the availability of telehealth services [6].

Telehealth has been particularly beneficial for people with cancer during the COVID-19 pandemic due to increased risk of adverse outcomes from infections in immunocompromised patients [8]. A scoping review of the use of telehealth technology in people with cancer during the COVID-19 pandemic included 29 studies from across the USA, the UK, Italy, Australia, Saudi Arabia, China and Canada [9]. The results of the review showed that various telehealth technology including video conferencing, telephone consultation and messaging applications were being widely used by people with cancer for teleconsultation, televisit, telerehabilitation and telemonitoring [9]. The study showed positive outcomes in terms of users’ satisfaction with the telehealth services [9]. While an Australian study of nearly 40,000 people accessing cancer services showed that those who used telehealth services had higher odds of emergency department (ED) visits and unplanned hospitalisation [10], whether these outcomes differ between people with and without cancer or with other chronic diseases and the sociodemographic and comorbidity characteristics associated with telehealth service use remains to be explored [11]. Identifying predictors associated with higher healthcare service use among people with cancer compared with the general population and quantifying the extent of differences in health status by healthcare service and cancer status could help direct resources and inform planning of future service development. Leveraging technology to access healthcare, particularly for high-need groups, could help improve continuity of care and health outcomes [3].

To address these gaps in knowledge, the aims of this study were to compare the following: (i) the prevalence and characteristics associated with telehealth service use in people with and without cancer, (ii) self-reported health status, visits to ED and hospitalisation by telehealth usage and cancer status and (iii) perceptions of care among those who used telehealth services in people with and without cancer.

## Methods

### Data source

We used data from the Patient Experience Surveys (PES) conducted by the Australian Bureau of Statistics (ABS) for the last 2 financial years (2023–2024 and 2022–2023) in random samples (generated based on computer algorithm) of people aged ≥ 15 years who were usual residents of private dwellings throughout Australia [12, 13]. Data were collected from participants via telephone interviews about their experiences with a range of healthcare services in the past 12 months before the interview, including general practitioners, specialists, hospitals, ED and telehealth services. Data were collected from 26,176 people in 2023–2024 PES and 25,934 people in 2022–2023 PES. We accessed the de-identified data for analysis through the ABS secure DataLab [14].

### Study population

We identified people who self-reported of having cancer as one of the long-term health conditions as the cancer group and the remaining participants as the non-cancer group. We restricted our study population to people aged ≥ 25 years as the healthcare needs in adolescents and young adults (described as 15–24 years in Australia) with cancer may be distinct from those in adults [15].

### Telehealth services

Participants were asked if they had used a telehealth service (i.e. appointments with a health professional over the phone, by video conferencing or through other communication technologies) for their own health in the last 12 months, and the types of health professionals they had the telehealth services with. For those who had a telehealth service, their perceptions of the service were collected in the PES using a 5-point scale (always, often, sometimes, rarely or never), including if all the telehealth practitioners they had seen: (1) listened carefully to them; (2) showed respect for what they had to say; (3) spent enough time with them. For the purpose of this study, we grouped the responses into two categories as ‘always/often’ and ‘sometimes/rarely/never’ to meet minimum cell count requirements for data privacy (i.e. no cell contained fewer than 10 observations). Lastly, participants were also asked if they would use telehealth again for consultation (yes or no) if it was offered. For those who had used a telehealth service but were not capable of answering for themselves due to injury, illness or language barriers (i.e. interviews that were conducted by proxy), they were not asked these questions related to their perceptions of the service.

### Sociodemographic and other long-term health conditions

Sociodemographic information including sex, age (grouped as < 50, 50–65, 65–79 and ≥ 80 years); country of birth (Australia, main English-speaking countries, others); geographical location (major cities or others); marital status (married or not married); employment status (employed or unemployed/not in labour force); highest education level (postgraduate, bachelor’s degree, diploma, certificate or no non-school qualification/missing data); socioeconomic status (measured by the index of relative socioeconomic disadvantage 2016) [16] and the private health insurance status were extracted.

Participants were asked whether they have any of the following individual long-term health conditions that have lasted, or are likely to last, for 6 months or more: (1) arthritis or osteoporosis, (2) asthma, (3) diabetes, (4) heart or circulatory condition, (5) mental health condition including depression or anxiety, (6) long-term injury or (7) any other long-term health conditions.

### Self-assessed health status, and use of ED services and hospitalisations

Self-assessed health status was collected in the PES whereby participants were asked ‘In general, would you say that your health is excellent, very good, good, fair or poor?’ We grouped the responses into two categories: (1) good health comprising rating of excellent, very good or good health status, and (2) poor health comprising rating of fair or poor health status [17].

We also analysed the data on whether the participants had been to a hospital ED (yes or no) and had been admitted to hospital (yes or no) in the past 12 months.

### Statistical analysis

Descriptive analysis was conducted to summarise the characteristics of the study population by telehealth service use and cancer status. We conducted separate multivariable logistic regression analyses to examine the characteristics associated with the use of telehealth service in the overall study population, those with cancer, and those without cancer. Characteristics considered were sociodemographic factors (sex, age, country of birth, geographical location, marital status, employment status, education level, socioeconomic status and private health insurance status), presence of selected individual types of long-term health conditions, and the year of PES (2023–2024 or 2022–2023). The results were reported as adjusted odds ratio (aOR) with 95% confidence intervals (CIs).

We also compared self-assessed health status, use of ED services and hospitalisations by telehealth service use and cancer status using multivariable logistic regression. The models were adjusted for sociodemographic factors, presence of selected individual types of long-term health conditions and year of PES as described above.

For those who had a telehealth service and provided opinions on their perceptions of the service, descriptive analysis was performed to compare their responses between people with and without cancer.

All analyses were conducted using R version 4.4.1.

### Ethics approval

This study was approved by the Flinders University Human Research Ethics Committee Low Risk Panel (#7218) and was performed in accordance with the ethical standards of the 1964 Declaration of Helsinki and its later amendments.

## Results

### Cohort characteristics

A total of 47,547 people aged ≥ 25 years from across the two PES were included in the cross-sectional analysis (Table 1). Of these, 2109 people were identified as having a cancer and the remaining 45,438 people as the non-cancer group. Over one-third of people with cancer (*n* = 797, 38%) and one-quarter of people without cancer (*n* = 11,984; 26%) had a telehealth service in the past 12 months. The proportion of people with telehealth was higher in the 2022–23 PES than the 2023–24 PES in both cancer (40% vs 35%) and non-cancer groups (28% vs 24%). About half of the people who had a telehealth service were aged < 50 years in the non-cancer group and were aged 65–79 years in the cancer group, with similar age distribution between those with and without telehealth service in their respective cancer and non-cancer counterparts.

**Table 1:** Characteristics of the study population by cancer status

Characteristics, *n* (%)	Cancer			Non-cancer			*p*-value^a^
	Total *n*= 2109	Telehealth *n*= 797	No telehealth *n*= 1312	Total *n*= 45,438	Telehealth *n*= 11,984	No telehealth *n*= 33,454	
Sex							
Female	1019 (48)	402 (50)	617 (47)	24,763 (55)	7797 (65)	16,966 (51)	< 0.001
Male	1090 (52)	395 (50)	695 (53)	20,675 (45)	4187 (35)	16,488 (49)	
Age group, years							
< 50	148 (7)	65 (8)	83 (6)	20,559 (45)	5545 (46)	15,014 (45)	< 0.001
50–64	483 (23)	197 (25)	286 (22)	11,360 (25)	2992 (25)	8368 (25)	
65–79	1044 (49)	401 (50)	643 (49)	10,350 (23)	2739 (23)	7611 (23)	
≥ 80	434 (21)	134 (17)	300 (23)	3169 (7)	708 (6)	2461 (7)	
Country of birth							
Australia	1578 (75)	622 (78)	956 (73)	30,366 (67)	8720 (73)	21,646 (65)	< 0.001
Main English-speaking	262 (12)	96 (12)	166 (13)	5017 (11)	1285 (11)	3732 (11)	
Others	269 (13)	79 (10)	190 (14)	10,055 (22)	1979 (16)	8076 (24)	
Geographical location							
Major cities	1254 (60)	451 (57)	803 (61)	29,757 (66)	8088 (68)	21,669 (65)	< 0.001
Others	855 (40)	346 (43)	509 (39)	15,681 (34)	3896 (32)	11,785 (35)	
Marital status							
Married	1104 (52)	443 (56)	661 (50)	25,316 (56)	6611 (55)	18,705 (56)	0.001
Not married	1005 (48)	354 (44)	651 (50)	20,122 (44)	5373 (45)	14,749 (44)	
Employment status							
Employed	514 (24)	216 (27)	298 (23)	27,759 (61)	7245 (61)	20,514 (61)	< 0.001
Unemployed/not in labour force	1595 (76)	581 (73)	1014 (77)	17,679 (39)	4739 (39)	12,940 (39)	
Highest education level							
Postgraduate	207 (10)	94 (12)	113 (9)	6084 (13)	1983 (17)	4101 (12)	< 0.001
Bachelor’s degree	283 (13)	130 (16)	153 (12)	9185 (20)	2658 (22)	6527 (20)	
Diploma	222 (11)	102 (13)	120 (9)	4751 (11)	1330 (11)	3421 (10)	
Certificate	448 (21)	163 (20)	285 (22)	9236 (20)	2288 (19)	6948 (21)	
No non-school qualification/missing	949 (45)	308 (39)	641 (49)	16,182 (36)	3725 (31)	12,457 (37)	
Socioeconomic status							
Decile 1–2	471 (22)	178 (22)	293 (22)	8316 (18)	2054 (17)	6262 (19)	< 0.001
Decile 3–4	468 (22)	175 (22)	293 (22)	8838 (20)	2188 (18)	6650 (20)	
Decile 5–6	416 (20)	160 (20)	256 (20)	9492 (21)	2546 (21)	6946 (21)	
Decile 7–8	390 (19)	150 (19)	240 (18)	9723 (21)	2666 (22)	7057 (21)	
Decile 9–10	364 (17)	134 (17)	230 (18)	9069 (20)	2530 (21)	6539 (19)	
Private health insurance status							
Yes	1248 (59)	505 (63)	743 (57)	27,314 (60)	7773 (65)	19,541 (58)	< 0.001
No	861 (41)	292 (37)	569 (43)	18,124 (40)	4211 (35)	13,913 (42)	
Long-term health condition							
Arthritis/osteoporosis	1054 (50)	423 (53)	631 (48)	10,742 (24)	3596 (30)	7146 (21)	< 0.001
Asthma	331 (16)	129 (16)	202 (15)	4956 (11)	1986 (17)	2970 (9)	< 0.001
Diabetes	332 (16)	126 (16)	206 (16)	3604 (8)	1150 (10)	2454 (7)	< 0.001
Heart/circulatory condition	788 (37)	315 (40)	473 (36)	8190 (18)	2712 (23)	5478 (16)	< 0.001
Mental health condition	453 (22)	200 (25)	253 (19)	7785 (17)	3489 (29)	4296 (13)	< 0.001
Long-term injury	373 (18)	161 (20)	212 (16)	4949 (11)	1863 (16)	3086 (9)	< 0.001
Other long-term condition	606 (29)	280 (35)	326 (25)	8854 (20)	3451 (29)	5403 (16)	< 0.001
Survey year							
2022–2023	1051 (50)	423 (53)	628 (48)	22,597 (50)	6431 (54)	16,166 (48)	< 0.001
2023–2024	1058 (50)	374 (47)	684 (52)	22,841 (50)	5553 (46)	17,288 (52)	
Types of telehealth professionals	N/A		N/A	N/A		N/A	
General practitioner		589 (74)			10,194 (85)		< 0.001
Medical specialist		358 (45)			2266 (19)		< 0.001
Nurse		54 (7)			476 (4)		< 0.001
Other health professionals		48 (6)			1022 (9)		< 0.001

^a^Comparisons across four groups (cancer with telehealth, cancer without telehealth, non-cancer with telehealth, and non-cancer without telehealth) for all characteristics, except for types of telehealth professionals with comparisons across two groups (cancer with telehealth and non-cancer with telehealth)

A higher proportion of people used telehealth services was born in Australia (73–78% versus 65–73%) and had a higher education level (61–69% versus 51–63% with a non-school qualification), private insurance (63–65% versus 57–58%) and a long-term health condition (e.g. 25–29% versus 13–19% with a mental health condition) compared to people without a telehealth service in both cancer and non-cancer groups. The most common telehealth professional seen by those with a telehealth service was a general practitioner (74% in cancer versus 85% in non-cancer), followed by a medical specialist (45% in cancer versus 19% in non-cancer).

### Characteristics associated with telehealth service use

Several characteristics were associated with a higher likelihood of having had a telehealth service in the past 12 months including presence of cancer (aOR = 1.62, 95% CI = 1.46–1.78) and all the individual types of long-term health conditions (aOR ranged from 1.32 for diabetes to 2.21 for mental health condition) examined in the study (Table 2). Characteristics associated with a lower likelihood of telehealth service were being male, older age, unmarried, unemployed, uninsured, completing the PES survey in a more recent year (i.e. 2023–2024), having been born overseas, resided outside of major cities, and a lower education level. These similar trends were observed in both the overall study population and the non-cancer group.

**Table 2:** Characteristics associated with telehealth service use (yes versus no (reference))

Characteristics	Adjusted odds ratio (95% CI)		
	Overall	Cancer	Non-cancer
Sex			
Female	Reference	Reference	Reference
Male	0.61 (0.58–0.64)*	0.84 (0.69–1.02)	0.60 (0.57–0.63)*
Age group, years			
< 50	Reference	Reference	Reference
50–64	0.82 (0.77–0.87)*	0.92 (0.62–1.36)	0.81 (0.76–0.86)*
65–79	0.79 (0.74–0.86)*	0.87 (0.58–1.31)	0.78 (0.72–0.84)*
≥ 80	0.61 (0.55–0.68)*	0.65 (0.41–1.03)	0.61 (0.54–0.68)*
Country of birth			
Australia	Reference	Reference	Reference
Main English-speaking	0.86 (0.81–0.93)*	0.92 (0.69–1.22)	0.86 (0.80–0.93)*
Others	0.63 (0.60–0.67)*	0.64 (0.48–0.86)*	0.64 (0.60–0.68)*
Geographical location			
Major cities	Reference	Reference	Reference
Others	0.85 (0.81–0.90)*	1.19 (0.98–1.45)	0.84 (0.80–0.88)*
Marital status			
Married	Reference	Reference	Reference
Not married	0.89 (0.85–0.93)*	0.80 (0.66–0.97)*	0.90 (0.86–0.94)*
Employment status			
Employed	Reference	Reference	Reference
Unemployed/not in labour force	0.93 (0.88–0.99)*	0.89 (0.68–1.15)	0.93 (0.87–0.99)*
Highest education level			
Postgraduate	Reference	Reference	Reference
Bachelor’s degree	0.82 (0.76–0.88)*	1.00 (0.69–1.45)	0.81 (0.76–0.88)*
Diploma	0.74 (0.98–0.80)*	0.99 (0.67–1.46)	0.72 (0.66–0.79)*
Certificate	0.64 (0.59–0.69)*	0.66 (0.46–0.94)*	0.64 (0.59–0.69)*
No non-school qualification/missing	0.56 (0.52–0.60)*	0.58 (0.41–0.80)*	0.56 (0.52–0.60)*
Socioeconomic status			
Decile 1–2	0.95 (0.88–1.03)	1.23 (0.89–1.70)	0.94 (0.87–1.01)
Decile 3–4	0.96 (0.89–1.03)	1.20 (0.88–1.63)	0.94 (0.88–1.01)
Decile 5–6	1.05 (0.98–1.13)	1.14 (0.84–1.56)	1.05 (0.98–1.12)
Decile 7–8	1.03 (0.97–1.10)	1.16 (0.86–1.58)	1.03 (0.96–1.10)
Decile 9–10	Reference	Reference	Reference
Private health insurance status			
Yes	Reference	Reference	Reference
No	0.78 (0.74–0.82)*	0.80 (0.65–0.98)*	0.78 (0.74–0.81)*
Long-term health conditions			
Arthritis/osteoporosis			
No	Reference	Reference	Reference
Yes	1.33 (1.25–1.40)*	1.21 (0.99–1.48)	1.35 (1.27–1.43)*
Asthma			
No	Reference	Reference	Reference
Yes	1.50 (1.41–1.60)*	0.91 (0.70–1.18)	1.55 (1.45–1.65)*
Diabetes			
No	Reference	Reference	Reference
Yes	1.32 (1.22–1.42)*	1.03 (0.79–1.33)	1.36 (1.25–1.47)*
Heart/circulatory condition			
No	Reference	Reference	Reference
Yes	1.48 (1.40–1.57)*	1.21 (0.99–1.48)	1.51 (1.42–1.60)*
Mental health condition			
No	Reference	Reference	Reference
Yes	2.21 (2.09–2.33)*	1.27 (1.01–1.60)*	2.27 (2.14–2.40)*
Long-term injury			
No	Reference	Reference	Reference
Yes	1.41 (1.32–1.50)*	1.21 (0.94–1.55)	1.43 (1.34–1.53)*
Other long-term conditions			
No	Reference	Reference	Reference
Yes	1.79 (1.70–1.88)*	1.63 (1.33–2.00)*	1.80 (1.71–1.90)*
Survey year			
2022–2023	Reference	Reference	Reference
2023–2024	0.78 (0.75–0.82)*	0.79 (0.66–0.94)*	0.78 (0.75–0.81)*
Cancer status No Yes	Reference 1.62 (1.46–1.78)*	NA	NA

*p-value < 0.05

For the cancer group, the presence of mental health and other long-term health conditions was associated with a higher likelihood of telehealth service use, whereas being unmarried, uninsured, having a lower education level (i.e. certificate or no non-school qualification) and completing the PES survey in a more recent survey were associated with a lower likelihood of telehealth service use. The results were non-significant for other variables in the cancer group.

### Self-assessed health status, and use of ED services and hospitalisations

**Fig. 1:**
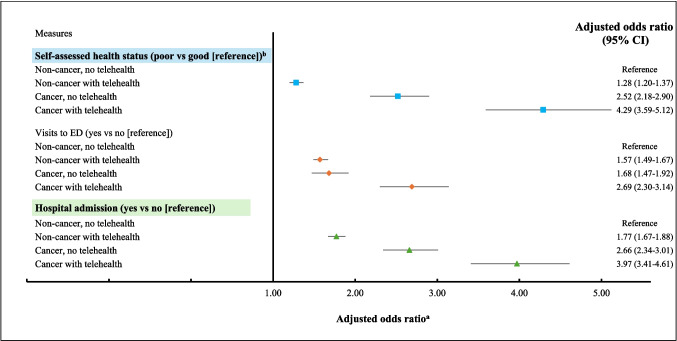
Comparison of self-reported health status, visits to emergency departments and hospitalisation by cancer status and use of telehealth. ^a^Adjusted for sociodemographic (sex, age, country of birth, geographical location, marital status, employment status, education level, socioeconomic status, private health insurance status), presence of individual types of long-term health conditions and year of survey. ^b^Good health comprising rating of excellent, very good or good health status and poor health comprising rating of fair or poor health status

Relative to people without cancer and telehealth use, the odds of reporting poor health status was highest in people with cancer and telehealth use (aOR = 4.29, 95% CI = 3.59–5.12). This was followed by people with cancer who did not use telehealth (aOR = 2.52, 95% CI = 2.18–2.90), and people without cancer who had a telehealth service (aOR = 1.28, 95% CI = 1.20–1.37) (Fig. 1).

The odds of having had an ED visit and a hospital admission in the past 12 months followed these similar trends.

### Perceptions of telehealth service use

Among those who had a telehealth service and responded to the questions related to their perceptions of telehealth, the majority of them (86–88%) reported that they would use telehealth again in both cancer and non-cancer groups (Table 3). The majority of people who had a telehealth service reported that the telehealth practitioners always/often listened carefully to them (93–95%), showed respect for what they had to say (95%), and spent enough time with them (91–93%) in both cancer and non-cancer groups. There were no significant differences between cancer and non-cancer groups in their responses to those questions.

**Table 3:** Perceptions of telehealth service

Description, *n* (%)	Cancer *n* = 721	Non-cancer *n* = 11,303	*p*-value
Would use telehealth again			
Yes	620 (86)	9991 (88)	0.060
No	101 (14)	1312 (12)	
Telehealth practitioners listened carefully			
Always/often	686 (95)	10,529 (93)	0.046
Sometimes/rarely/never	35 (5)	774 (7)	
Telehealth practitioners showed respect			
Always/often	686 (95)	10,706 (95)	0.680
Sometimes/rarely/never	35 (5)	597 (5)	
Telehealth practitioners spent enough time			
Always/often	670 (93)	10,293 (91)	0.101
Sometimes/rarely/never	51 (7)	1010 (9)	

## Discussion

This cross-sectional study showed a higher prevalence of telehealth service use in people with cancer compared to people without cancer. A higher telehealth use in the cancer group may indicate a higher burden of disease and healthcare needs among people living with cancer. Two factors, including the presence of mental health conditions and other long-term health conditions, were associated with a higher odds of telehealth service use in people with cancer, whereas four other factors including being unmarried, uninsured, having a lower education level and completing PES survey in a more recent year were associated with a lower odds of telehealth service use. Relative to people without both cancer and telehealth use, the odds of reporting poor health status, having had an ED visit and a hospital admission in the past 12 months were highest among people with cancer who used telehealth, followed by people with cancer who did not use telehealth and people without cancer who had a telehealth service. Overall, people who used telehealth showed positive perceptions of the service regardless of cancer status.

Studies using data from the 2022 Health Information National Trends Survey showed that 39–43% of US adults received care from a health professional using telehealth in the past 12 months [18–20], which was higher than the estimates found in our study ranging from 26% in the non-cancer group to 38% in the cancer group. This difference may reflect the types of study population (cancer versus general population) across studies, patient or provider preferences, literacy and access to digital technology and complexity of care across study population [21]. Our study also demonstrated that the proportion of people who had a telehealth service decreased over time in both cancer (from 40% in 2022–23 to 35% 2023–24 survey) and non-cancer groups (from 28 to 24%), similar to that observed in a study among US adult general population (from 37% in 2021 to 30% in 2022) [22] and in oncology practices (33% in 2020 then levelled off to 12–15%) [21] which may be due to patient preferences of returning to in-person care options as the pandemic restrictions eased [23]. While over one-third of the cancer group in our study received telehealth service, the majority did not. Barriers to accessing telehealth services especially in these group of patients with high burden of disease warrant further investigation [24].

Comparable to prior findings [18, 19], the presence of long-term health conditions was identified as predictors of telehealth service use in our study. As comorbidities contribute to a significant healthcare burden, the use of telehealth provides a feasible care option for management of select chronic diseases which have been shown to improve patient outcomes [25] and may also reduce the financial burden associated with time, travel and costs [26]. Our study showed that the presence of mental health conditions, in particular, was associated with significantly higher odds of telehealth service in people with cancer. This result was consistent with the findings of a study conducted in Florida, USA, in the Medicaid beneficiaries which showed that patients needing psychiatric care were highly likely to use telehealth service, albeit in the non-cancer population [27]. Taken together, the findings suggest there may be continued opportunities to integrate telehealth for those who have complex healthcare needs and/or require regular service use to increase access to care.

Our study found several factors including being unmarried, uninsured, having a lower education level and a more recent survey year to be associated with lower odds of telehealth service use in both cancer and non-cancer groups. While telehealth service is covered by the Australian Government’s Medicare Benefits Schedule, it is possible that some Australian residents would have access to the direct-to-consumer telehealth services operating outside of publicly funded Medicare system [28]. Out-of-pocket costs could be a barrier to assessing such service in those without a private health insurance. The two PES were conducted between July 2022-June 2023 and July 2023-June 2024, which may have contributed to differences in findings by survey year in relation to the timing of COVID-19 restrictions. Our findings of the effects of sociodemographic factors including marital status and education level on telehealth service use were consistent with prior studies [20, 29]. Other characteristics associated with decreased odds of telehealth service observed in the general population included older age and male [18, 19, 30, 31], comparable to our findings in the non-cancer group although the results for these variables did not reach statistical significance in the cancer group which may be due to relatively modest sample size. Considerable disparity exists in internet access and digital skills among older adults which may affect their ability to use telehealth services [32]. It is plausible that men may generally be less likely to engage with medical services compared to women due to decreased health seeking behaviours associated with traditional masculine stereotypes [33]. In contrast, other studies found telehealth use was higher among males than females [34, 35], and the gender differences observed across studies may partly reflect variation in disease burden, health-seeking awareness and technology literacy. Investing resources into educating older patients and those with lower level of education attainment and promoting positive masculinity in the context of health and the use of technologies in their care may help improve use of telehealth services in these populations.

The odds of reporting poor health status, having had an ED visit and a hospital admission in the past 12 months were highest in people with cancer and telehealth use, followed by people with cancer who did not use telehealth and people without cancer who had a telehealth service, when compared to people without cancer and telehealth usage. People with cancer had higher healthcare needs [36] and poorer health status [17] than those without cancer and common reasons for presentation to ED and hospitalisation included side effects associated with cancer treatment, infections and cancer disease progression [37–39]. The relationship between telehealth and other health services use appears to be bidirectional. It is plausible that the presence of selected conditions such as cancer prompts presentation to ED and that leads to the introduction of virtual ED as part of the emergency telehealth services [40]. Urgent telehealth visits could also lead to more presentations to ED and some conditions might require urgent physical examinations and/or diagnostic assessment at ED or in the hospital settings following telehealth consultation where remote care could not be delivered effectively [30]. A systematic review included 127 randomised controlled trials examining the effect of telehealth on hospital services found that while small to moderate reductions in hospital service use could be achieved through telehealth, there were uncertainties surrounding the magnitude of the effects as not all differences reached statistical significance [41]. Further longitudinal study is required to examine the effects of telehealth on other health service utilisation in people with cancer and other chronic diseases to identify which aspects of telehealth result in positive and negative outcomes.

Reassuringly, over 90% of people who used telehealth in our study showed positive perceptions of the service regardless of cancer status. Several other studies have also described positive themes surrounding patient satisfaction with telehealth service [42, 43]. Taken together, these findings suggest that telehealth could be a feasible option in healthcare delivery and that the integration of telehealth into cancer models of care to complement in-person care could provide a hybrid approach combining the benefits of both [24, 44]. Further longitudinal study to investigate the effects of telehealth on health outcomes including clinical and patient-reported outcomes is needed to inform the development of health policy and strategies that promote an innovative approach to healthcare delivery.

This study has several limitations. Self-reported data was used in this study which may be subject to recall and response bias [45, 46]. As information on the date of services was not collected in the PES, we were unable to differentiate the sequences of telehealth service in relation to visits to the ED and hospitalisation. We were also not able to perform further subgroup analysis by type of cancer and time from cancer diagnosis and by frequency of telehealth service use as the information was not available in the dataset. It is possible that there may be lower engagement in the surveys among selected populations such as people from culturally and linguistically diverse backgrounds [47]; selected questions in the PES such as perceptions of telehealth service were not asked among those with language barriers. Nonetheless, given that there is limited data on telehealth in people with cancer compared to the general population, our study provided useful insights into the telehealth use in this cohort of people.

## Conclusion

While over one-third of people with cancer utilised a telehealth service, the majority did not. A higher odds of ED presentation and hospitalisation in people with cancer who had a telehealth use may reflect greater care needs. Further research should investigate the barriers to accessing telehealth services in these group of people with high disease burden to inform the development of health policy and strategies targeting people with the greatest needs.

## Data Availability

The data may be accessed through the Australian Bureau of Statistics with the appropriate approvals.
